# Energetics of Cardiac Blood Flow in Hypertrophic Cardiomyopathy through Individualized Computational Modeling

**DOI:** 10.3390/jcdd10100411

**Published:** 2023-09-27

**Authors:** Owen Baenen, Angie Carolina Carreño-Martínez, Theodore P. Abraham, Sandra Rugonyi

**Affiliations:** 1 Department of Mechanical Engineering, Rice University, Houston, TX 77005, USA; owen.baenen@gmail.com; 2 Biomedical Engineering Department, Oregon Health & Science University, Portland, OR 97239, USA; 3 USCF HCM Center, Division of Cardiology, Department of Medicine, University of California San Francisco, San Francisco, CA 94158, USAtheodore.abraham@ucsf.edu (T.P.A.)

**Keywords:** hemodynamics, patient-specific modeling, computer simulation, heart function, hypertrophic cardiomyopathy, cardiac flow, kinetic energy, vorticity

## Abstract

Hypertrophic cardiomyopathy (HCM) is a congenital heart disease characterized by thickening of the heart’s left ventricle (LV) wall that can lead to cardiac dysfunction and heart failure. Ventricular wall thickening affects the motion of cardiac walls and blood flow within the heart. Because abnormal cardiac blood flow in turn could lead to detrimental remodeling of heart walls, aberrant ventricular flow patterns could exacerbate HCM progression. How blood flow patterns are affected by hypertrophy and inter-patient variability is not known. To address this gap in knowledge, we present here strategies to generate personalized computational fluid dynamics (CFD) models of the heart LV from patient cardiac magnetic resonance (cMR) images. We performed simulations of CFD LV models from three cases (one normal, two HCM). CFD computations solved for blood flow velocities, from which flow patterns and the energetics of flow within the LV were quantified. We found that, compared to a normal heart, HCM hearts exhibit anomalous flow patterns and a mismatch in the timing of energy transfer from the LV wall to blood flow, as well as changes in kinetic energy flow patterns. While our results are preliminary, our presented methodology holds promise for in-depth analysis of HCM patient hemodynamics in clinical practice.

## 1. Introduction

Hypertrophic cardiomyopathy (HCM), characterized by a thickening or hypertrophy of the heart’s left ventricular (LV) wall, is one of the most common inherited heart diseases, affecting about 1 in 400 people of all ages [1,2]. In HCM, heart morphology is altered, leading to high blood pressure gradients across the LV outflow tract (LVOT) in 2/3 of the patients (obstructive HCM, or oHCM) and no impact on LVOT pressure gradients in the other 1/3 (non-obstructive HCM, or nHCM). The severity and location of the wall thickening impact LV blood flow dynamic patterns [3] and may influence clinical presentation and disease progression [4]. HCM alters the heart anatomy, impacts cardiac function, and can lead to heart failure, arrhythmias, and even sudden cardiac death [2,5,6].

Since there is substantial inter-person variability in HCM hypertrophy patterns, the impact on LV blood flow dynamics is highly individualized. Blood flow dynamics plays an essential role in regulating cardiac tissue remodeling and hypertrophy, and thus contributes to HCM progression. Moreover, the interaction of blood and the cardiac walls involves a cyclic energy exchange that regulates cardiac performance [7]. The dynamics of blood flow within the heart ventricles is therefore linked to heart morphology and function and can either enhance or diminish cardiac efficiency and the progression of hypertrophy. Yet the details of HCM ventricular hemodynamics have been understudied. This is in part due to a lack of time-efficient and accurate methods to quantify cardiac blood flow. In this study, we present a methodology we are developing to bridge the gap in accurately and efficiently quantifying ventricular hemodynamics in individual HCM patients using image-based computational simulations of blood flow dynamics. To demonstrate the capabilities of our developed strategy, we applied it to three patients (one normal, one nHCM, and one oHCM). We then compared simulated data from the three patients, finding marked differences in hemodynamic parameters that could potentially be used to classify HCM patients and individualize treatment in the future.

### 1.1. Hemodynamics and Cardiac Tissue Remodeling

Blood flow interacts with cardiac tissues, generating hemodynamic forces that act on cardiac cells within tissues. These forces, mainly from blood pressure and the friction exerted by blood flow through wall shear stresses, are sensed by cells and elicit tissue responses that can alter normal cardiac tissue composition and morphology through cardiac remodeling processes [8,9,10,11]. For example, increased blood pressure leads to thickening of cardiac walls and hypertrophy, while increased wall shear stress in blood vessels leads to vessel dilation [8,9,12,13,14]. There is still much to be learned about how hemodynamics and the energetics of blood flow dynamics within the heart affect cardiac function and the continuous remodeling of the heart walls, including the expansion of hypertrophic regions in HCM.

### 1.2. Cardiac Imaging 

Current imaging technologies, such as cardiac magnetic resonance (cMR) imaging and echocardiography (Echo), allow for the visualization and analysis of the heartbeating dynamics [4,15,16]. Together, cMR and Echo technologies have allowed a deeper understanding of HCM clinical presentation, disease progression, and the identification of patients at risk for complications. Nevertheless, the analysis of blood flow within the heart remains challenging. This is because the heart beats fast, the heart chambers deform considerably during the cardiac cycle, and blood flow exhibits complex behavior within the heart ventricles, including flow rotation and vortex formation, which are difficult to measure accurately [17]. Thus, analysis of cardiac efficiency is typically based on blood flow velocities measured as blood enters or exits the heart as well as cardiac wall motion, which, for example, allows quantification of ejection fraction, stroke volume, and wall strain. These analyses are limited as they do not provide the details of blood flow within the heart and hence cannot accurately assess abnormal energetic losses due to pathological flow dynamics or hemodynamic forces exerted on cardiac walls. While 4D flow cMR is emerging as a way of quantifying intracardiac blood flow velocity fields [18,19], it is expensive, requires additional scanning time (compared to acquiring regular cMR), and has limited accuracy. To counteract these limitations, computational fluid dynamics (CFD) simulations that are based on patient dynamic images (typically cMR [20]) have been employed [17,21,22,23,24]. 

### 1.3. Our Approach: Cardiac Digital Twins—Challenges and Limitations

We describe here a patient-specific, image-based approach to simulate and quantify the individualized dynamics of blood flow within the heart LV. To exemplify the approach capabilities, we simulated the blood flow dynamics within the LV of a normal heart and two hearts with HCM: (1) an oHCM case in which the LV wall thickening is such that interferes with normal ejection of blood to the aorta by partially obstructing blood flow and generating an increased pressure gradient in the LVOT; and (2) a mild nHCM case. We expect simulations will enable a better understanding of LV flow energetics that will potentially enhance diagnosis and prognosis in HCM and beyond. 

Image-based CFD uses the motion of the heart walls extracted from cardiac images and physics-based models to simulate the flow of blood within the heart [17,21]. While these cardiac CFD models (also called patients’ heart digital twins) are becoming more common in studies of heart function and cardiac disease, significant limitations in the generation of these computational models still exist. These limitations, mainly in steps that extract the dynamic cardiac anatomy from images and transfer this information to CFD software, have hindered hemodynamic analyses on a large scale. As a result, most studies involve only a handful of cases. The approach presented here is a step in streamlining CFD model generation from patient images by generating semi-automatic procedures that shorten the time needed for CFD model generation while preserving model reproducibility and accuracy. We also briefly compare 2D and 3D CFD models regarding accuracy and ease of implementation. We further discuss the limitations and implications of our findings and how to further improve our methods to aid patients.

### 1.4. Significance 

The potential clinical benefits of evaluating heart function through detailed CFD modeling are significant. Image-based CFD can enhance existing imaging technologies by allowing quantification of parameters previously unavailable (e.g., wall shear stresses, vortex formation [23,25]) or only available through invasive procedures or estimates (such as pressure gradients). Patient-specific, image-based models of the heart (or cardiac digital twins) could also aid in patient risk assessment, treatment decision-making, and planning stages for surgical procedures [17,21]. 

## 2. Materials and Methods

We used human cMR images to develop a digital twin of the heart LV that simulates the flow of blood within the ventricle. Human images were available as part of a database of HCM patients treated since 2017 at the University of California San Francisco (UCSF) HCM Center of Excellence, one of the largest clinical HCM programs in the US. Prior to the study, images were de-identified, and the use of patient images was approved under UCSF IRB # 20-30348. We selected cMRs from three patients: (1) a patient with a normal heart; (2) a patient with nHCM; and (3) a patient with oHCM. The patients’ diagnosis was determined clinically and was part of their medical records. We describe below the procedures implemented to extract the heart geometry and heart wall motion over the cardiac cycle from cMR images, as well as the methods employed to develop patient-specific CFD models used to simulate cardiac blood flow dynamics within the heart LV. 

### 2.1. Cardiac Magnetic Resonance (cMR) Images and Image Segmentation

For each patient, a set of cMR images was available and used for individualized CFD model development. The set consisted of phase contrast cine cMR images of long-axis and short-axis views of cardiac sections, with 20 images per cardiac cycle per section. The short-axis view sections spanned the entire heart. The long-axis views generally showed two or three sections of the heart that were approximately perpendicular to the short-axis sections. Overall, the set of images allows identification of hypertrophic (thickened) portions of the LV walls and their effect on cardiac wall motion.

For CFD model development, we used the patient’s cMR images to reconstruct the geometrical volume of the heart’s LV lumen. To this end, we used short-axis and long-axis view cMR images in Digital Imaging and Communication in Medicine (DICOM) format, a standardized imaging format that contains metadata useful for reconstruction and image organization. Overall, cMR images encompassed distinct sections (long and short views) and cardiac cycle phases (20 images depicting the cardiac cycle). We started by delineating (segmenting) from the various cMR images the LV lumen–wall interface.

**Figure 1:**
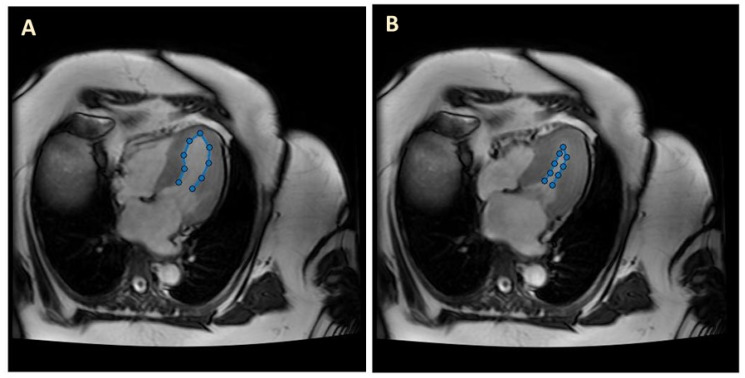
Example cMR long-axis images and LV segmentation (blue). (**A**) End diastole (ED); (**B**) end systole (ES).

LV image segmentation was performed using an in-house custom-made MatLab program (MathWorks Version R2022a). The MatLab code extracted the traces of the segmented LV lumen–wall interface from each individual image. For long-view sections, we used a manual segmentation procedure to perform the delineations (see Figure 1). After manual tracing of the lumen–wall interface, the MatLab program simply exported the trace coordinates for further processing (e.g., spatiotemporal smoothing) and data organization in preparation for CFD model generation.

Manual segmentation is extremely time-consuming and subject to user variability and fatigue. To improve the accuracy and reproducibility of the segmentation task and shorten the time to model generation for 3D models, we implemented a semi-automatic segmentation procedure using MatLab. For short-view sections, LV segmentation was performed using an unsupervised Fuzzy C Means method [26,27], followed by a deformable active contour (snake) model [28], which corrected (if needed) and smoothed the initial segmentation. Our unsupervised Fuzzy C Means method assigns a membership probability to each point among clusters using the reciprocal distance of the set of pixels from cluster centers. Cluster centers are updated according to a formula involving fuzzy memberships and the pixel intensity histogram. The method minimizes the following objective function:

(1) $$J=\sum_{i=1}^{N}\sum_{i=1}^{C}{\left(u_{ij}\right)}^{m}{\left\|q_{i}-c_{j}\right\|}^{2}$$

where *N* is the number of data points (image pixels); *C* is the number of cluster centers; *u_ij_* is the membership probability of the *i*th pixel, *q_i_*, to the *j*th cluster; *m* is the fuzzy weight exponent, *m* ∈ (1, ∞); *c_j_* is the center of the *j*th cluster; and ‖*q_i_* − *c_j_*‖ represents the distance between the *i*th pixel and the *j*th cluster center. 

**Figure 2:**
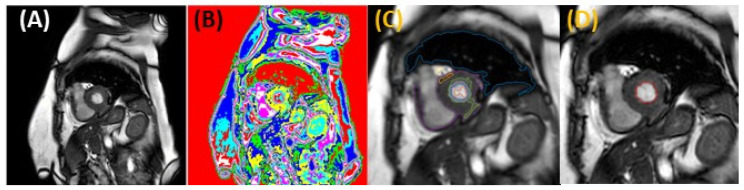
Example LV segmentation from a short-axis view. (**A**) Sample original contrasted image. (**B**) Cluster image representation. Each unique color refers to a different cluster (c = 10). (**C**) Image with closest traces to ventricle center from each cluster (enlarged). (**D**) Computed best fit trace for the region of interest, the LV (enlarged).

Before clustering, anisotropic diffuse filtering was applied to all images to reduce the influence of false lines. Ten clusters were partitioned for the short-view image data sets. The optimal cluster number was determined by minimizing the mean inter-cluster distance from cluster centroids while retaining relative circular cluster symmetry within the LV (short views). Cluster centers were estimated by sampling the intensity range and selecting uniform intervals within that range. The best-fit cluster that included the ventricle lumen region boundaries was determined by finding the largest area region amongst clusters that were closest to a manually approximated ventricle center for each view, provided prior to clustering through user input (by selecting a center point in a representative image). Minimal morphological closing was applied to smooth the resulting region and minimize irregularities. The obtained lumen region was then delineated by tracing the lumen–wall interface (see Figure 2). LV delineations were refined by identifying and eliminating non-target traces in each model (e.g., removing traces with a large deviation from circularity and expected ventricle location in short views). Traces were then used to initialize a snake -deformable contour model. The snake model uses gradients of intensity from the images to correct the position of the lumen–wall interface (when needed) and minimize the strain energy of the trace, which smooths the contour. Final smoothing of traces was implemented to avoid surface irregularities in the reconstruction by simply averaging neighboring corresponding trace points in space. Using the *ImagePosition* and *ImageOrientation* attributes of the DICOM files, 2D delineations from image sections were used to obtain a 3D reconstruction of the LV. 

While we aimed at automating the segmentation task, the algorithm employed (described above) is semi-automatic, limiting its applicability to larger studies. In our implementation of the segmentation algorithm, an expert user evaluates the automatically generated traces from the clustering algorithm (the Fuzzy C Means method) and makes manual corrections when needed. These corrections are mostly minor (a small adjustment to the trace or additional trace smoothing), but occasionally traces miss the LV—lumen–wall interface (~5% of traces for the algorithm used in this manuscript). In these cases, the lumen–wall interface is re-traced manually by the user, and the manual trace is smoothed using active contour algorithms. We are actively working on introducing algorithmic changes to avoid trace inaccuracies while increasing the efficiency of the segmentation task. 

### 2.2. CFD Models of the LV

To simulate LV blood flow over the cardiac cycle, we used finite element methods (FEM) and the FEM commercial software ADINA 9.8 (Bentley Systems, Inc., Exton, PA, USA). Using ADINA and the traces obtained from segmentations of the cMR images, we generated CFD models with moving boundaries that mimicked the patient’s motion of the LV lumen–wall interface. Within the FEM model, the LV geometry was discretized with a finite element mesh (a grid of points or nodes that are connected by elements). Since the LV lumen boundaries move over time, expanding during diastole and contracting during systole, each mesh element changes its shape to accommodate the LV lumen change. An arbitrary Lagrangian–Eulerian (ALE) formulation was employed within ADINA CFD to correctly account for mesh motion. As simulations of the LV contraction and expansion proceeded, a sequence of corresponding meshes that tracked the LV motion was generated.

We created two distinct CFD models of the LV that were based on cMR patient images: (1) 2D models that capture the LV wall motion obtained from long-view planes; and (2) 3D models that incorporate the 3D motion of the LV wall reconstructed from short-view planes. While the procedures employed in generating these two models are similar, 3D models are more involved than 2D models, so we will describe model generation separately.

All simulations and models used the same hemodynamic parameters. The heart rate was assumed to be 60 beats per minute, or 1 beat per second, for all 3 patients studied. Blood flow viscosity was μ = 4 cP, and blood density was ρ = 1060 kg/m^3^. Blood was assumed to be Newtonian, and the dynamics of blood flow was modeled using the transient Navier–Stokes equation. Moreover, according to cMR imaging data, the systolic phase encompassed about 40% of the cardiac cycle, while diastole accounted for 60% of the cycle for all 3 patients considered.

#### 2.2.1. Two-Dimensional Image-Based Model of the LV

We started by modeling the LV in 2D, assuming planar flow within ADINA. We used the LV traces manually extracted from long-view patient cMR images (see Figure 1) to generate the 2D model lumen geometry and its change over the cardiac cycle. Our 2D CFD models included the motion of the LV wall below the aorta and mitral valve, with blood flow driven by changes in the LV lumen area. The LV lumen–wall interface was modeled as a moving boundary, and blood flow was modeled using an ALE formulation with the fluid domain discretized using quadrilateral elements. In our implementation, to impose boundary motion (moving wall), we used the fluid–structure interaction (FSI) feature in ADINA: the wall was modeled as a 2D line of structural beam elements with imposed displacements (applied at all nodes) that simulated the motion of the lumen–wall interface; this wall structural motion was coupled to the CFD model of the lumen. The open end of the ventricle (close to the aorta outlet and mitral inlet) was assigned a zero traction (approximately zero pressure) boundary condition, so that blood could enter and exit the heart through this boundary. Thus, the open end of the LV acts as both the heart inlet and outlet (IO), depending on the phase of the cardiac cycle considered (diastole vs. systole, respectively). By applying a zero-traction boundary condition to the IO boundary, the 2D CFD model solves for gradients of pressures (with respect to the IO) within the LV, which are useful to compute the exchange of force/work between the LV wall and blood. 

**Figure 3:**
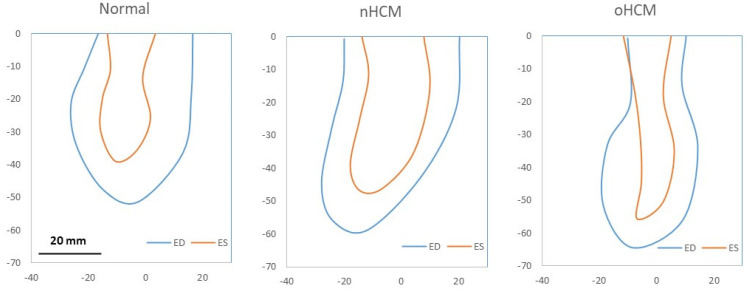
LV traces from the 3 cases studied. The figure shows traces at end diastole (ED, blue) and end systole (ES, orange) for 3 hearts: a normal heart, a heart with non-obstructive HCM (nHCM), and a heart with obstructive HCM (oHCM). The top boundary (y = 0) is the inlet-outlet (IO) boundary for each model. All three plots are on the same scale, and the scale bar is 20 mm (shown on first plot only).

LV traces from the 3 patients employed in models spanned the cardiac cycle, including systolic and diastolic phases (see Figure 3) and a range of LV wall motion that reflected the patient-specific HCM (or normal) anatomy. While cMR images include 20 time steps spanning the heart-changing geometry during the cardiac cycle, in this study, we only traced 8 times. These time steps were selected as follows: end diastole (ED), end systole (ES), and two additional steps in between during systole, as well as 4 additional time steps during diastole. A linear interpolation was applied between traces to model additional time steps for simulations. 

Simulations of the 2D CFD models quantified the blood flow velocity vector field, **v**(**x**, *t*) = **v**(*x*, *y*, *t*), within the 2D LV lumen (*x*, *y* coordinates), as well as blood pressure differences with respect to the LV IO boundary. To further quantify blood flow energy distributions, we computed the flow kinetic energy density (*KE*):

(2) $$KE(x,t)=\frac{1}{2}\rho {\left\|v\right\|}^{2}$$

To compare the energetics of blood flow among the 3 hearts, we further computed the spatial *KE* average ($\bar{KE}$) as follows:

(3) $$\bar{KE}\left(t\right)=\frac{1}{A}\iint KE\;dA$$

where *A* is the area of the LV lumen from the 2D model. 

The vorticity vector (**Ω**) is defined as the rotor of the velocity field (**v**),

(4) Ω(x,t)=∇×v

In 2D, the vorticity vector, which is perpendicular to **v**(*x*, *y*, *t*), is normal to the flow plane (**Ω** = Ω**_z_ e**_z_), with **e**_z_ a unit vector in the out-of-plane direction (i.e., *z*-direction), simplifying visualization and analysis of **Ω**. Depending on the velocity gradient and swirling of the flow, the vorticity could be positive or negative. This allows us to compute a vorticity magnitude average ($\bar{\left\|Ω\right\|}$), and a vorticity average ($\bar{Ω}$) as follows:

(5) $$\bar{\left\|Ω\right\|}(t)=\frac{1}{A}\iint \left\|Ω\right\|\;dA$$

(6) $$\bar{Ω}\left(t\right)=\frac{1}{A}\iint {Ω}_{z}\;dA$$

where $\bar{Ω}$ represents the balance of vorticity in the LV (as a perfectly symmetric LV should render $\bar{Ω}$ = 0), given the assumptions of the model. 

Finally, a useful and relatively new parameter that can be computed from cardiac images or CFD computations is the hemodynamic force [29,30], **F**(*t*), defined as the total force exchanged between the blood and the cardiac wall. Mathematically,

(7) $$F\left(t\right)=\iint \sigma \cdot n\;dS$$

where σ is the flow stress tensor, and **n** is a unit vector normal to the LV lumen–wall boundary, *S*. Expressed in a Cartesian coordinate system (*x*, *y*, *z*) in 3D, (*x*, *y*) in 2D, the components of the stress tensor, $\sigma_{ij}$, are

(8) $$\sigma_{ij}=-p_{ii}+\mu \left(\frac{\partial v_{i}}{\partial x_{j}}+\frac{\partial v_{j}}{\partial x_{i}}\right)$$

where *p* is the hydrostatic pressure, $v_{i}$ are the components of the velocity vector, and $x_{i}$ the components of the position vector (**x**). **F**(t) is dominated by pressure forces, although wall shear stresses also contribute. In our implementation, **F**(t) is obtained from reaction forces in the ADINA CFD model. 

We compared hemodynamic parameters among the 3 dynamic 2D CFD models of the LV that we generated.

#### 2.2.2. Three-Dimensional Image-Based Model of the LV

**Figure 4:**
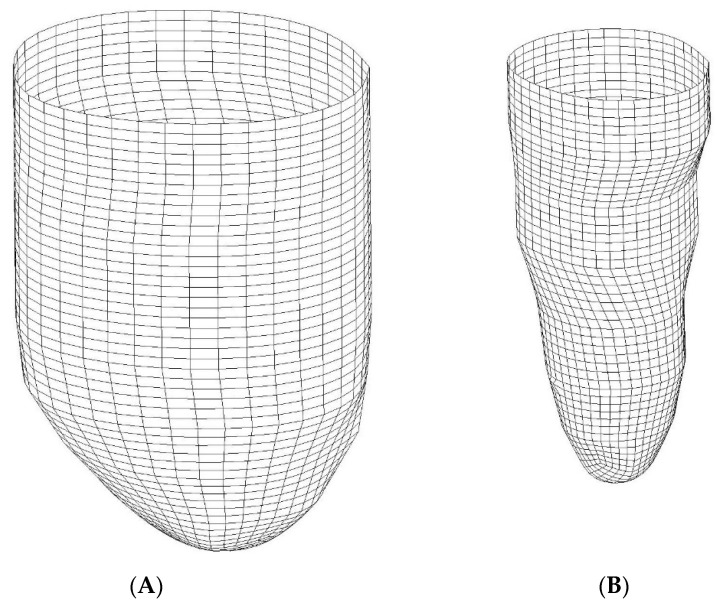
Example of corresponding meshes used to impose wall motion in LV models. (**A**) End diastole (ED); (**B**) end systole (ES). By applying displacements to each node, the model simulates the LV wall motion during systole (ED to ES) and diastole (ES to ED).

We also modeled the LV in 3D using ADINA. To this end, we used the LV traces extracted from short-view patient cMR images using our developed semi-automatic segmentation algorithm (see Figure 2) and reconstructed the lumen–wall surface in space (e.g., see Figure 4). Here, we used reconstructions from ED and ES and linear interpolation between these two LV geometries to simulate the systolic and diastolic phases of the cardiac cycle. To this end, the LV lumen–wall interface was modeled as a 3D moving shell surface with imposed displacements (using FSI in the ADINA software). Like 2D models, our 3D CFD models included the motion of the LV wall below the aorta and mitral valve, with blood flow driven by changes in LV lumen volume and inlet/outlet flow through the open end of the LV model, the IO surface. 

A difficulty in generating 3D models was creating registered and corresponding surface meshes at ED and ES. Corresponding meshes have the same number of nodes and elements, such that applying displacements (translation movement) to each node on the lumen–wall interface shell surface, we can describe the LV contraction and expansion during the cardiac cycle. Correspondence was obtained by generating a surface grid that was consistent among the ED and ES phases using mesh generation tools in ADINA, ensuring that the same number of nodes at corresponding locations was generated (see Figure 4). For instance, the apex portion of the heart LV was meshed using 4 triangular curved shells, and the portion above was meshed with a rectangular cylindrical grid. Mesh generation for both meshes started at corresponding locations and followed the exact same procedures to further ensure mesh correspondence. 

Volumetric meshes for the CFD model were also generated using ADINA mesh generation tools. We discretized the CFD lumen volume using tetrahedral elements and used the ALE formulation to account for mesh deformation over time that simulated the contraction and expansion of the LV.

Simulations of the 3D CFD models quantified blood flow velocity vector field, **v**(**x**, *t*) = **v**(*x*, *y*, *z*, *t*), within the LV lumen, as well as blood pressure differences with respect to the LV IO surface. Using the velocity field, we computed the volume flow rate (*Q*) through the LV IO surface as

(9) $$Q\left(t\right)=\iint v\cdot n\;dA$$

where **n** is a unit vector normal to the IO surface, and *A* here represents the area of the IO surface. 

We then proceeded to compute the stroke volume (SV), which is the total volume of blood delivered to the body during the cardiac cycle and can be computed simply as the difference between the LV volume at ED and ES, or alternatively as the time integral of *Q*(*t*) during systole (from ED to ES). 

As with 2D models, we quantified the flow kinetic energy density (*KE*) following Equation (2). We also calculated the rate of energy or power (*P*) of blood exiting or entering the heart as

(10) $$P\left(t\right)=\iint KE(v\cdot n)\;dA$$

We compared hemodynamic parameters among the 3 patient heart models considered. 

## 3. Results

Two- and three-dimensional LV models solved for the velocity and pressure fields within the LV lumen and allowed comparison of hemodynamic parameters among the three patients considered. Clinical cardiac measurements are listed in Table 1. Because flow in our models is driven by the motion of the lumen–wall interface (traced from cMR images), simulations of the CFD models reveal how the interaction between the LV cardiac wall and blood determines blood flow. 

### 3.1. Two-Dimensional Individualized CFD Models of the LV

**Figure 5:**
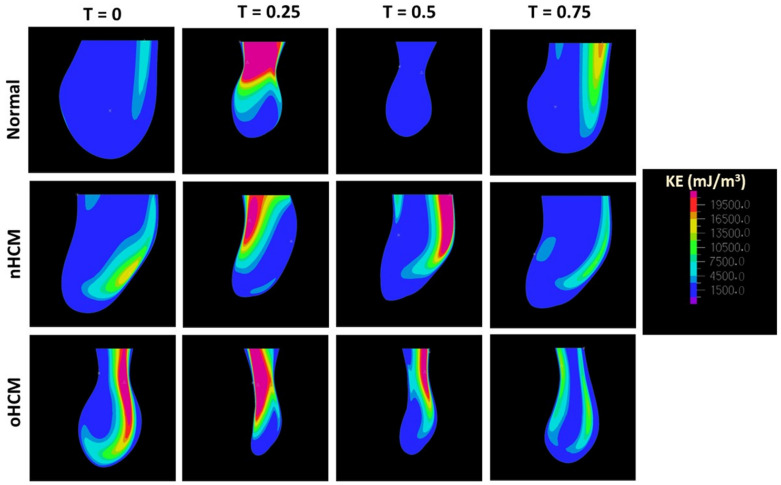
Comparison of the spatial distribution of kinetic energy (KE) density at different time points over the cardiac cycle among different patients. Columns: T indicates normalized cardiac cycle period (from 0 to 1), with 0 corresponding to end diastole (ED). The second column (T = 0.25) corresponds to systolic ejection, and the third and fourth columns (T = 0.5 and 0.75) correspond to diastolic filling. Rows: Results from three patients are shown. nHCM: non-obstructive HCM; oHCM: obstructive HCM. Plots are color-coded by kinetic energy (KE) density values.

Two-dimensional models are relatively simple and easy to set up and simulate, while also providing a general depiction of blood flow within the LV. Because 2D models neglect out-of-plane flow, the velocity field **v**(*x*, *y*, *t*) can be readily visualized. Plotting the distribution of *KE* (see Equation (2)) in space and time, *KE*(*x*, *y*, *t*), provides a visual map of blood flow energy within the LV during systolic contraction and diastolic relaxation (see Figure 5). This is also an estimated measure of the energy transferred from the contractile heart walls to the blood to circulate blood through the entire body, as well as flow energy from the left atrium that enters the LV during relaxation. An efficient heart will use most of the transferred energy to push blood to the body, with minimal energy loss (energy dissipation) within the LV. In the normal heart, maximum *KE* occurs during the systolic phase (T = 0.25) when blood is ejected to the body through the aorta. In contrast, the HCM hearts exhibit portions of flow with increased *KE* during diastole (T = 0.5 and T = 0.75). Simulation of 2D models captures flow asymmetries during systole and diastole (Figure 5) that are driven by the cardiac wall motion. 

**Figure 6:**
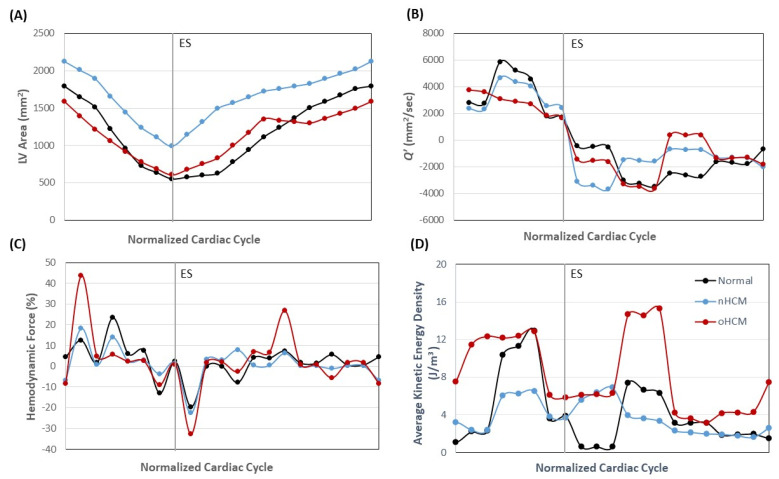
Simulation results obtained from 2D LV models for normal, nHCM, and oHCM patients. The plots show the variation of (**A**) LV area; (**B**) hemodynamic force; (**C**) flow rate (*Q*′); and (**D**) average kinetic energy density over the cardiac cycle. The vertical gray line indicates end systole (ES), and the beginning and end of the plot correspond to end diastole (ED).

**Figure 7:**
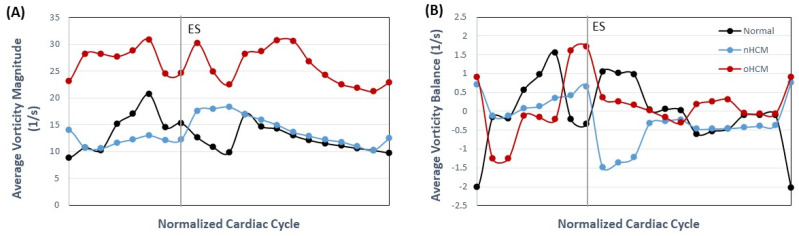
LV blood flow vorticity from 2D models. (**A**) Average vorticity magnitude over the cardiac cycle depicts the magnitude of the vorticity vector (perpendicular to the longitudinal plane) averaged over the LV long-view surface. (**B**) Average vorticity accounting for the vorticity vector direction (positive or negative) to determine whether vorticity is balanced.

To better compare heart function for the three cases over the cardiac cycle, we computed overall and average quantities (see Figure 6 and Figure 7) and plotted hemodynamic parameters over time. The cardiac cycle was normalized (depicted from T = 0 to 1), and plots start and end at ED, when the heart is most expanded. Plots first depict the systolic phase that culminates with ES, followed by the diastolic phase. For 2D models, we started by calculating the LV area (in the 2D plane) over time during the cardiac cycle (Figure 6A), as captured from cMR image segmentations. These area plots allow us to compare, for the longitudinal planes chosen, the dynamics of wall contraction and relaxation. HCM hearts seem to contract more uniformly during systole, while the control heart contracts slightly slower at the beginning and then more rapidly towards ES. HCM hearts also relax (fill) initially faster than the control heart during diastole, with the obstructive HCM heart exhibiting a short period of area decrease during diastole, which could indicate diastolic mitral regurgitation. This overall behavior is also reflected in plots depicting 2D flow rate, *Q*′(*t*), with *Q*′ exhibiting a short period of outward flow during diastole in the oHCM patient (Figure 6B). 

We have also compared the hemodynamic force (HDF) for the three models, computed as the vertical component of the force **F**(*t*), see Equation (7), which is the sum of reaction forces in the *y*-direction. HDF plots (Figure 6C) show a larger transfer of force from wall to blood in the oHCM case during systole, reflecting the extra effort of the cardiac walls to push blood flow through an obstructed aorta (even though the aorta was not modeled). Meanwhile, the nHCM heart closely replicated the HDF dynamics of the control heart during systole (albeit at slightly different levels) but was out of synchrony with the control heart during diastole. Comparisons of average *KE* (Equation (3)) over the cardiac cycle clearly show two peaks, one in systole and one in diastole, for the control heart (see Figure 6D). In the nHCM heart, the systolic peak is less prominent, while the diastolic peak occurred earlier than in the control heart and corresponds to a faster than normal initial volume (area) recovery early during diastole. In the oHCM heart, in contrast, the systolic peak is longer, indicating an overall increased transfer of energy to the blood and energy dissipation (with respect to the normal heart), as well as higher flow energy during the systolic phase, perhaps pointing to the lower efficiency of the oHCM heart in pumping blood. This is also reflected in the increased average vorticity magnitude (Equation (5)), which indicates increased energy dissipation, of the oHCM heart (see Figure 7A). Meanwhile, the vorticity in the normal and nHCM hearts is more comparable, although the nHCM vorticity is out of phase with respect to that of the normal heart. The average vorticity balance (Equation (6)) further shows the lack of symmetry of the LV motion and thus lumen blood flow (see Figure 7B).

### 3.2. Three-Dimensional Individualized CFD Models of the LV

**Figure 8:**
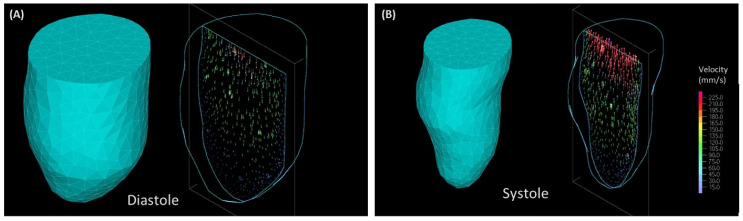
Patient-specific CFD modeling of HCM. Three-dimensional LV geometry and velocity vectors at a cut plane during diastole (**A**) and systole (**B**) for an nHCM patient. Velocity vectors (right plots of each panel) are color-coded by magnitude.

**Figure 9:**
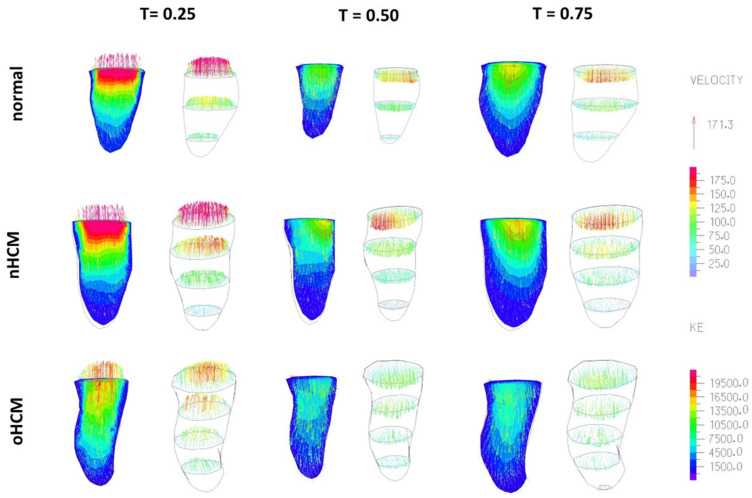
Comparison of simulation results from cMR-based CFD modeling of the LV. Three patient-specific models are shown: (**top**) a normal heart; (**middle**) a heart with non-obstructive HCM (nHCM); and (**bottom**) a heart with obstructive HCM (oHCM) at three phases during the cardiac cycle, with T indicating normalized (0–1) cardiac cycle period. T = 0.25 corresponds to systole, while T = 0.5 and T = 0.75 correspond to diastolic filling. Each pair of images shows blood velocities (in mm/s), represented by arrows. The left image of the pairs also shows the flow kinetic energy (*KE*, in mJ/m^3^) at long-view cut planes (color coded by value), while the right image shows blood flow velocity vectors at short-view planes (color-coded by magnitude). Model depictions are to scale, with the distance between short-view (cross-sectional) planes equal to 16 mm.

Simulation of 3D models of the LV for the three patients considered (see Figure 8 and Figure 9) computed the blood flow dynamics during systolic ejection and diastolic relaxation. Analysis of simulation results confirmed similar stroke volume for the normal and nHCM patients (45 mL and 59 mL, respectively), and reduced stroke volume (34 mL) for the oHCM patient. The rate of energy (power, *P*; see Equation (10)) carried by the blood exiting the LV during systole is considerably reduced in the oHCM patient: the maximum exiting flow energy rate during systole is about 3 mW for both the normal and nHCM patients, and about 0.5 mW for the oHCM patient. Within the limitations of our 3D models, which interpolate geometries between ED and ES, these results highlight the distinct LV blood flow dynamic patterns in HCM subtypes. 

**Figure 10:**
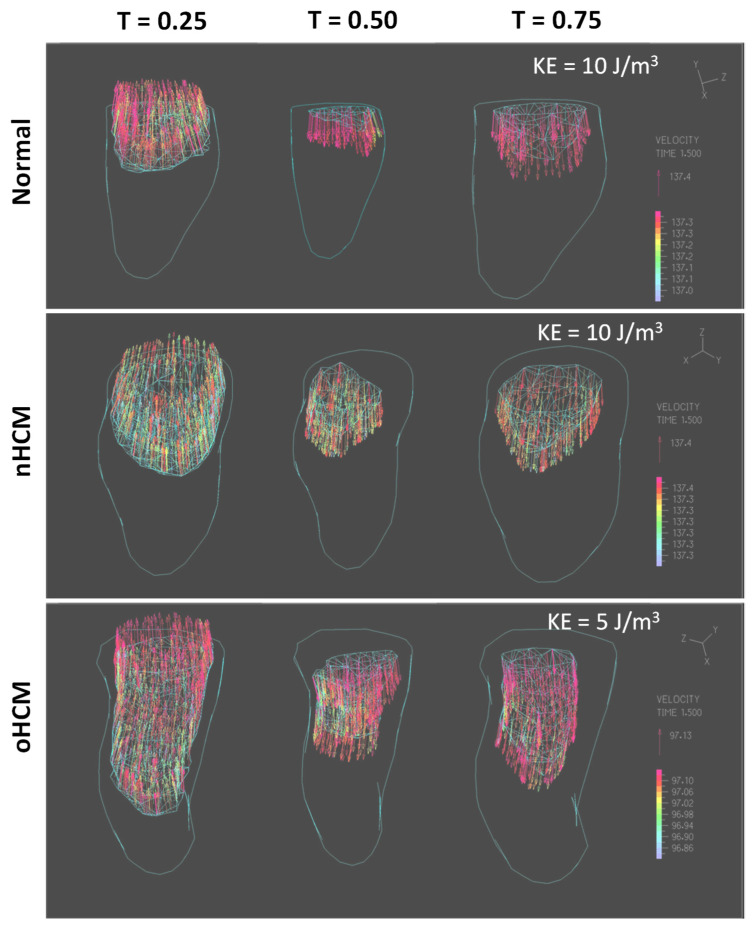
Simulation results for the three patients considered depicting the kinetic energy density (KE) and flow fields. The distinct graphs depict velocity vectors within the LV inside the volume of a KE isosurface (such that KE > isosurface value of 10 J/m^3^ or 5 J/m^3^). Three phases of the cardiac cycle (T ∈ [0,1]) are shown: T = 0.25 corresponds to systole, while T = 0.5 and T = 0.75 correspond to diastolic filling. Colors represent velocity magnitude, and the outer layer is the whole LV. Note that flow entering and leaving the heart is not symmetric but rather larger towards one side of the heart, particularly in the HCM hearts. Thus, natural ventricular flow asymmetry was partially captured by our models, even though our LV models were truncated and did not include the inlet and outlet within the heart base.

Three-dimensional models capture asymmetries in blood flow velocities and energy, especially during diastole (see Figure 9 and Figure 10). Because SV is significantly reduced in oHCM, and given that, in our models, flow exits the heart relatively uniformly over time during systole and enters the LV uniformly in time during diastole (due to linear interpolation between ED and ES), blood flow velocities and KE are significantly reduced in oHCM. Please note that this behavior was not highlighted by the 2D models, as the change in area for oHCM, while smaller than for the normal heart, was not as different as the change in volume (SV) in 3D due to the additional dimension considered. When looking at KE isosurfaces, which are 3D enveloping surfaces that share the same KE value (Figure 10), we can visualize asymmetries in blood flow (driven by the wall motion) as well as changes in blood flow dynamic trends.

## 4. Discussion

In clinical practice, the significant inter-individual variability of HCM presentation and disease course is well known and has been described [2,4,5,6,31,32]. A majority of HCM patients will live their lives with no significant restrictions or reduction in life span, but patients at risk develop serious and expensive complications. Moreover, patients in the same family carrying the same mutation could have very different anatomic and functional presentations. Importantly, disease course and susceptibility to developing complications such as heart failure and cardiac arrhythmias, including sudden death, also vary significantly among individuals. The variation in location and degree of hypertrophy and its hemodynamic consequences (e.g., presence or absence of LVOT obstruction) likely contribute to this variability. Even after accounting for physical and functional differences, risk prediction in HCM remains unreliable. A significant knowledge gap exists in how hypertrophy location and severity impact blood flow dynamics. CFD allows sophisticated interrogation of the efficiency of blood flow and generates hemodynamic parameters (e.g., flow kinetic energy, hemodynamic force) that are difficult or impossible to obtain from images alone. Our results, while preliminary (due to the small number of cases considered), show individual differences in hemodynamic parameters among HCM sub-types. We hope our methodology will lead to its application in larger groups of HCM patients to test whether CFD can improve the diagnosis of HCM sub-types and be used in predicting clinical outcomes over a longer period.

We present here a comparison of 2D and 3D CFD procedures based on cMR images to analyze cardiac blood flow within the LV of patients with HCM. Since only two HCM patients and one control subject were considered, our results cannot be used to inform clinical practice, but are key in getting insights into ways of quantifying the dynamics of blood flow within the heart with the goal of improving diagnosis and prognosis. 

It is worth noting that our CFD models simulated the motion of cardiac LV walls as extracted from cMR images, and hence, in our models, LV wall motion drives blood flow dynamics. In real life, the interaction between the cardiac walls and blood determines the dynamics of intracardiac blood flow and influences heart wall motion. Therefore, our computations represent an inverse modeling strategy in which we know the resulting wall motion and use it to simulate the ensuing dynamics of blood flow. Nevertheless, our study presents limitations that preclude full validation with patient data and could impact the interpretation of results. Such limitations are associated with our choice to use cMR imaging data, including temporal information used in models; assumptions regarding blood flow rheology, which deviate from the non-Newtonian behavior of blood; and the modeled LV blood flow domain, which missed morphological features from the heart base. Limitations and their consequences are discussed in more detail below. 

While the use of cMR images provides relatively good spatio-temporal resolution for our models, accuracy is limited to the imaging plane (with a pixel spacing of 0.7 mm). However, spacing between imaging planes (8 mm) somewhat limits the extraction of details, especially at the base of the heart, where the aortic and mitral valves are located. While we are currently working on addressing this limitation, in this study, the heart base was not included in our models. Instead, an IO boundary surface (3D) or line (2D) was introduced in models with no assumed asymmetries in flow inlet/outlet. Furthermore, 2D models were constructed from only one long view plane and 8 (out of 20 available) image time points (with linear interpolation in between). In contrast, 3D models reconstructed the LV lumen geometry from short-view planes and assumed linear interpolation between the ED and ES geometrical volumes when modeling systole and then diastole, neglecting the actual ejection and filling dynamics that occur during cardiac function. Thus, our CFD models do not account for asymmetric flow resulting from the off-center mitral inlet and aortic outlet [33,34] (see, e.g., Figure 8). Hence, any asymmetry in blood flow dynamics is purely due to the wall motion influenced in patients by the true dynamics of blood, which enters the heart through the mitral inlet on one side of the LV base and exits the heart through the aortic outlet on the other side of the LV base. It is therefore remarkable that flow asymmetries were captured by both 2D and 3D models of the LV and are indicative of a strong coupling between cardiac flow and wall motion. Nevertheless, these captured flow asymmetries should be interpreted with caution, as they are likely underestimated by our models. Acknowledging these limitations, the study in this paper is a steppingstone towards developing effective cardiac digital twins of the LV and a step in analyzing the pros and cons of 2D and 3D cardiac models. 

While 2D models are easier to implement, as they only require the motion of the wall on a single long-view plane, they are not accurate and can only be used to get a rough estimation of the effects of wall motion on LV blood flow dynamics. 2D models, moreover, are sensitive to the plane selected for imaging/segmentation as well as the accuracy of the wall motion segmentation. Nevertheless, given the ease with which 2D models can be implemented and the potential general insights to be gained from them, they are worth considering when contemplating CFD modeling on many patients, such as in cases of clinical trials. More research is required to determine when and to what extent 2D models are useful and can be predictive. For example, 2D models might prove useful in situations in which 3D data are not available or access to the data is difficult, such as retrospective ultrasound data acquired in 2D. For these cases, it is worth continuing to study similarities, differences, and synergies among 2D and 3D LV simulation data. 

Three-dimensional models of the LV are more involved than 2D models and require segmentation along different planes and time points over the cardiac cycle. These models are also sensitive to the quality of the segmentation and 3D LV geometry, especially as the LV walls expand and contract over the cardiac cycle. Care must be taken when accurately extracting and smoothing the LV wall motion (in space and time). This is typically time-consuming, and even nowadays, a ‘bottle neck’ in the implementation of 3D LV models. This difficulty results in many studies that, like ours, only consider a limited number of patients. Automation of procedures will certainly enable larger studies in the future. 

Considering limitations, our study shows that even relatively simple 2D and 3D models of the LV (that do not include the heart base) capture different blood flow dynamics in HCM sub-types and normal heart function. These diverse hemodynamic profiles are due to hypertrophy patterns affecting LV wall motion and are thus representative of the cases considered. Because the heart base was not included in models, certain features of the blood flow dynamics, such as diastolic and systolic vortices, were not captured, and thus the dynamic profile obtained is incomplete. The results presented are nevertheless indicative of the effects of wall motion on blood flow dynamics away from the heart base and could lead to a better understanding of the effects of HCM on ventricular flow, and how changes in wall motion (due to patient variability and HCM progression) affect flow independently of the detailed inlet/outlet flow dynamics. In this regard, the mismatch in energy transfer observed when modeling the normal heart and HCM sub-types (Figure 6C,D) in the 2D models points to cardiac inefficiencies. While an exact quantification of cardiac inefficiency is elusive, as the model is simplified, it might be enough to pinpoint severe vs. mild deficiencies in patients. Moreover, our simplified 3D models capture flow asymmetries that are solely due to LV wall motion, once again independent of asymmetric mitral inlet and aortic outlet flow dynamics and indicative of the efficiency with which the LV wall transfers energy to the blood during systolic contraction and relaxes during diastolic filling. Of course, no conclusions can be drawn from the results of only three cases, but our preliminary data are promising. A larger study outside of the scope of this paper is needed to determine the clinical utility of our simplified 2D and 3D models. Comparison with fully validated computational models, utilizing, for instance, ultrasound data, is also needed before we can draw conclusions. The goal of this manuscript is to present our methodology and its application to model cases, as well as show promising differences among HCM sub-types that might inform clinical practice in the future.

## 5. Conclusions and Final Remarks

Results and lessons learned from our efforts point in several directions to improve models. The most obvious are the inclusion of all time points available (20 from cMR images) in both 2D and 3D models and improving the efficiency of the segmentation algorithm. When undertaking these tasks, care must be taken to avoid artifactual oscillations in the model lumen area/volume that will produce erroneous flow dynamics (particularly during phases of slow or isovolumic expansion or contraction) when the LV is barely moving. Another improvement is to include the aortic outlet and mitral inlet, rather than having a common IO boundary, to properly capture the asymmetries in exit and entrance flow dynamics. Once again, implementing this strategy will require careful spatiotemporal segmentation of the heart geometry. This is particularly challenging for segmenting the base of the heart, as the cMR image plane distance (8 mm) leaves gaps in the visualization of the heart base 3D geometry and its motion over the cardiac cycle. Once implemented, however, 3D models complete with the heart base could accurately reflect the dynamics of blood flow within the LV and allow full validation against patient data (for example, Echo data measuring blood flow velocities through the aorta). Moreover, 3D simulations of blood flow dynamics in the LV can precisely capture the energetics of blood flow and force transfer between the cardiac wall and blood. This is an important consideration when trying to fully understand cardiac deficiencies and the toll of abnormal hemodynamics on cardiac wall remodeling.

Finally, for CFD strategies to be implemented in the clinic, methods for segmenting the LV geometry will need to become more automatic (and yet accurate), as they are currently the ‘bottle neck’ in the implementation of image-based modeling strategies, limiting the scope and applicability of studies. While CFD and image analyses have significantly improved in recent decades, efficient, accurate, and fast segmentation of patient data is still needed. As more accurate and efficient algorithms and models are developed, researchers will be able to undertake large-scale studies that will help us elucidate the reciprocal relationship between heart anatomy and blood flow dynamics as well as the role of ventricular blood flow in regulating HCM progression. This knowledge, in turn, will be useful for the diagnosis and prognosis of HCM and for developing improved treatment strategies for patients with HCM. As more engineering and clinical cardiology teams embark on these problems, the future is bright, and the development of fully automatic LV heart digital twins will undoubtedly allow clinicians to tackle complex diagnosis and prognosis and devise novel treatment strategies.

## Figures and Tables

**Table 1:** Clinical characteristics of the three patients considered. Data were obtained from a patient chart review. EF: ejection fraction; MR: mitral regurgitation; SAM: systolic anterior motion; SV: stroke volume.

Patient	EF	MR	SAM	SV [mL] ^1^
Normal	76%	no	no	45
nHCM	82%	no	yes	59
oHCM	65%	mild	yes	34

^1^ Based on 3D model volumes.

## Data Availability

The data presented in this study are available on request from the corresponding author. The data are not publicly available as it is based on patient images that need material transfer agreements (MTA) in place for sharing.
